# MOSQUITO EDGE: An Edge-Intelligent Real-Time Mosquito Threat Prediction Using an IoT-Enabled Hardware System

**DOI:** 10.3390/s22020695

**Published:** 2022-01-17

**Authors:** Shyam Polineni, Om Shastri, Avi Bagchi, Govind Gnanakumar, Sujay Rasamsetti, Prabha Sundaravadivel

**Affiliations:** 1 STEM Enhancement in Earth Sciences, NASA Center for Space Research, Austin, TX 78723, USA; polineni.shyam@gmail.com (S.P.); omshastri@gmail.com (O.S.); avibagchi32@gmail.com (A.B.); govind.gnanakumar@gmail.com (G.G.); mosquitoedge@gmail.com (S.R.); 2 Department of Electrical Engineering, The University of Texas at Tyler, Tyler, TX 75702, USA

**Keywords:** mosquito prediction, edge sensing frameworks, citizen science

## Abstract

Species distribution models (SDMs) that use climate variables to make binary predictions are effective tools for niche prediction in current and future climate scenarios. In this study, a Hutchinson hypervolume is defined with temperature, humidity, air pressure, precipitation, and cloud cover climate vectors collected from the National Oceanic and Atmospheric Administration (NOAA) that were matched to mosquito presence and absence points extracted from NASA’s citizen science platform called GLOBE Observer and the National Ecological Observatory Network. An 86% accurate Random Forest model that operates on binary classification was created to predict mosquito threat. Given a location and date input, the model produces a threat level based on the number of decision trees that vote for a presence label. The feature importance chart and regression show a positive, linear correlation between humidity and mosquito threat and between temperature and mosquito threat below a threshold of 28 °C. In accordance with the statistical analysis and ecological wisdom, high threat clusters in warm, humid regions and low threat clusters in cold, dry regions were found. With the model running on the cloud and within ArcGIS Dashboard, accurate and granular real-time threat level predictions can be made at any latitude and longitude. A device leveraging Global Positioning System (GPS) smartphone technology and the Internet of Things (IoT) to collect and analyze data on the edge was developed. The data from the edge device along with its respective date and location collected are automatically inputted into the aforementioned Random Forest model to provide users with a real-time threat level prediction. This inexpensive hardware can be used in developing countries that are threatened by vector-borne diseases or in remote areas without cloud connectivity. Such devices can be linked with citizen science mosquito data platforms to build training datasets for machine learning based SDMs.

## 1. Introduction

Mosquitoes are one of the world’s most dangerous organisms, spreading deadly diseases like malaria, Dengue, and Zika. They have spread to nearly every continent and are further increasing their range due to the extreme weather conditions caused by climate change [1]. The ability to identify mosquito hotspots (areas of high probability of mosquito presence) can be especially valuable in preventing the spread of mosquitoes and the diseases they carry [2].

Due to the information revolution, we are now capable of custom manufacturing circuit boards (PCBs), i.e., more specialized boards akin to Raspberry Pi and Arduino that are well suited for certain endeavors over others. Compared to their satellite counterparts, such edge devices are significantly less expensive both to build and deploy. This makes them ideal for use in areas in which there is minimal existing infrastructure. Rather than deploying a network of satellites, we simply deploy a network of edge devices, collecting massive troves of data on their localized climates using external sensors. With such large amounts of data, we are capable of making real time predictions of greater accuracy.

**Figure 1:**
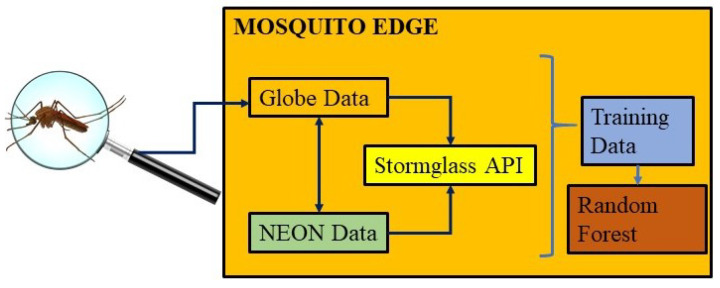
Overview of the proposed method in MOSQUITO EDGE framework.

The notion that mosquitoes pose a significant threat to humans across the world is the primary basis from which the underlying question of this study arises: How can climate and citizen science mosquito data be used to develop a machine learning algorithm that can predict mosquito hotspots? This question aims to shed light on a new perspective towards understanding and predicting potential mosquito hotspots by analyzing climate variables (such as temperature, climate cover, precipitation, humidity, and water vapor) as key determinants of species’ habitats. This technique combines older work done with species distribution models with Random Forest methods to increase accuracy. If an accurate mosquito threat prediction model is created and deployed on the edge at a low cost using completely autonomous technology, underdeveloped areas can be more easily prepared for mosquito-borne disease outbreaks. Our device would be able to connect with other devices (when possible), to form a highly linked system with a small energy footprint and high predictive capability. More importantly, this end-to-end process is built around consideration for the user and provides a reliable way for any individual to understand the mosquito threat near them, as well as the sensor data used to determine that threat. Not only are users capable of accessing that sensor data, but they are also able to modify and manually verify it as necessary. Figure 1 provides the overview of the proposed methodology.

## 2. Literature Review

### 2.1. Species Distribution Models

Species distribution models (SDMs) use climate variables and species observations to project the niche of an organism [3]. In the onset of the centralization of ecological data, species data are often used as a binary “presence” and “absence” variable for efficiency and ease of analysis [4]. This has popularized the use of AI-powered binary classification models in niche prediction. Popular models include CART, logistic regression, neural networks, Naive Bayes, and Maxent [5]. However, an approach with the label as a categorical variable does present challenges, namely class imbalance [6]. Without a large amount of high quality absence data collected in the field, pseudo-absence points generated in the ecological background often outnumber the observed species presence points [7]. In classification models, this imbalance can skew predictions in favor of the most populous label [8]. In this study, the problem of class imbalance was avoided in two key ways. Firstly, quality absence data were allocated in an amount equal to the amount of presence data. Secondly, an edge computing device whose role is to identify and collect species data can balance observed presences with an equal number of pseudo-absences or even true absence observations taken at regular intervals. With the preliminary issue of class imbalance resolved, the second conundrum becomes choosing the ideal model. The two primary contenders are Maxent and Random Forests as deep learning is an area that has not been proven to be successful in niche projects [9]. Maxent is a maximum-entropy based model that is advantageous in that it only needs presence data and can adjust to continuous labels [10]. It generates pseudo-absences in a customized environmental background, allowing for efficient and often accurate projections free of class imbalance [11]. Random Forests, on the other hand, is sensitive to class imbalance [12] given its ensemble methods, but due to the aforementioned resolution of the class imbalance issue, this drawback need not be considered. Past literature has indicated that Random Forests is the most accurate in niche modeling because this ensemble approach enhances prediction power [13]. Given Random Forest’s compatibility with edge computing in the form of TensorFlow Lite, and in consideration of Maxent’s inability to be easily harnessed on external hardware, a Random Forests species distribution model was used.

To make use of these species distribution models, newer computing paradigms can be used for data collection and the running of the models. This way, niche prediction can be performed with low latency and high scalability through extending cloud computing abilities to allow computation to occur beyond the central network [14]. These computing paradigms in addition are stored through a diverse set of devices allowing us to use an Internet of Things (IoT) device capable of running SDMs. Citizen science has played a significant role in climate data collection and remote sensing by increasing the amount of locations and sources in which data are collected [15]. The crowdsensing of this collection has been made possible through the IoT service of mobile crowdsensing (MCS), which uses cloud centered architectures [16]. As this produces copious amounts of network traffic, mobile edge computing (MEC) has been suggested as a replacement that solves this problem by functioning on the network edge [16]. A form of crowdsensing combining elements of MEC and MCS is most beneficial for larger scale crowdsensing, such as global users inputting data regarding climate variables.

### 2.2. Edge Computing and Its Significance

As a result of a general increase in artificial intelligence services in use today, edge computing has been identified as a promising solution that shifts AI model training and prediction to the network edge [17]. Edge intelligence increases the operational efficiency of predictive decision-making and achieves low-latency data processing through shifting the computing capability to the network edge [17]. If a Hutchinson approach is to be used, a large database of climate variable values is required. Using edge intelligence over traditional data collection from a cloud data center can consume less bandwidth and make niche prediction perform at a higher level. In addition, energy consumed by the IoT devices is reduced by compressing sensed data and sending it to the edge [18].

### 2.3. AI for Climate Prediction

The use of climate variables and data in artificial intelligence can be extremely beneficial for a multitude of applications. From machine learning models that can perform disease prediction to habitat detection, several deep learning techniques have been utilized to advance the understanding of certain climate phenomena. For example, when detecting drought stress, computer vision has been found to be a useful machine learning tool [19]. Time-lapse image sequences of crops are combined with a transfer learning classification technique in order to perform image detection and classification [19]. When determining the climate variables to be used in a machine learning model, many factors must be taken into account. Historical data often show periods of disruption in the form of sudden extreme weather or natural disasters that could invalidate the relationship between these variables [20]. Therefore, certain care must be taken to accurately sample historical data. In order to account for drastic climate changes, there have been certain popular climate modeling tools for time series data. The auto-regressive integrated moving average model in particular has been extremely beneficial for analyzing time series data, with a common application being a forecasting model that utilizes several climate variables [21]. Along with this, researchers have used several other machine learning techniques such as Random Forest Regression, Support Vector Machines, and Hidden Markov models in order to predict the climate variables themselves [21]. This could be used in our study to identify the most important features for detection of mosquito presence. The Random Forest algorithm in particular has been used in several climate-based applications. This supervised learning makes use of a large amount of decision trees to perform both classification and regression [22]. In addition to predicting certain environments and performing forecast analysis, the use of climate variables in artificial intelligence can also be vital in predicting trends of diseases based on how they are transmitted. For example, research conducted in Malaysia utilized a k-means clustering algorithm in order to determine relationships between temperature and humidity and the spread of dengue [23].

To make niche predictions using data from several different geographical locations, data collection from a network of IoT devices must be optimized. IoT data collection methods can be reviewed based on the trade-offs between data accuracy and frequency of measurement requests [24]. Beyond the traditional concurrent data collection, trees are used in an updated single-hop network structure that efficiently determines how many time-slots are required for concurrent data collection [25]. This novel network framework works under the assumption that several IoT devices are “neighboring” one another and thus can be used to scale up research involving several geographic locations.

### 2.4. Internet of Things for Citizen Science

Utilizing Internet of Things (IoT) and edge computing presents a promising avenue to collect data effectively. Moreover, in utilizing edge computing over the cloud, information collected from citizen scientists can be processed and transferred far more efficiently. Furthermore, research done on utilizing citizen science data with an edge device to map information proved that such networks “have the potential to transform the roles of citizens” [26]. By utilizing citizen science data in tandem with edge computing, there are new avenues that can be opened for the mapping and tracking of data real time. For example, crowdsourcing data can be effectively transferred over time with the use of edge computing with faster relay speeds [27]. On a similar note, the developments within IoT are being used to help shape citizens to be more digitally apt, resulting in a smarter city overall. IoT has “proven to better sustain the sensing and connectivity, becoming thus an efficient tool to address a broader range of qualitative public services. Moreover, the innate versatility that citizen scientists provide can allow “for the field assessment of any events which makes citizens a more reliable source of information” [28]. Beyond urban cities and smart citizen implementation, IoT and citizen science data can be implemented in rural areas, where mosquitoes are most prevalent. Edge computing is a cost effective and user-friendly solution that can allow individuals experiencing the digital divide to interact with complex datasets [29]. Although citizen science and IoT has its benefits, it is important to consider “data protocol procedures” regarding privacy and analyze “user inputs to ensure data quality” [30]. In short, fully harnessing the potential of citizen scientists is a vital step towards the successful implementation of broader edge computing and IoT solutions.

### 2.5. Deep Learning Models in Species Classification

Remote sensing IoT technology will help predict mosquito outbreaks and prevent some of the million cases of mosquito-borne disease every year. An existing computer vision-enabled IoT device films mosquitoes and uses convolutional neural networks to identify Aedes and Culex mosquitoes [31]. While it exhibits high accuracy, it is limited to classifying mosquitoes between two species and does not account for overlapping object detection [31]. This feature can be improved upon in future work to include better edge computing and classification and more features can be developed to better predict areas of high mosquito density.

## 3. Methodology

### 3.1. Data Extraction

To construct the training dataset for an empirical species distribution model, both mosquito and climate data are needed. GLOBE Observer (an app under the oversight of the National Aeronautics and Space Administration) provides useful mosquito larvae abundance data obtained from mosquito traps built by citizen scientists. Location, date, and larvae abundance were extracted into a Pandas DataFrame through the GLOBE Observer API for Python [32]. Other variables concerning the mosquito trap itself were not considered in this study. Since our model is rooted on binary classification, the larvae abundance variable was converted into a binary, categorical variable. Sites with larvae abundances exceeding 25 larvae were classified as “infested” and assigned the location a label of “1”. Locations with larvae abundances below 25 were assigned “0”. About 2000 mosquito observations (latitude, longitude, date, and larvae abundance per observation) were exported.

To reinforce citizen science data with robust scientific data, mosquito observations sampled from carbon dioxide traps were appended to the GLOBE Observer data [33]. Technicians from the National Science Foundation’s National Ecological Observatory Network (NEON) collect data from these traps, providing us with reliable and abundant data. Once again, latitude, longitude, and date were recorded. NEON’s mosquito data were already binary (presence or absence), so no threshold was needed to record these mosquito observations. With the mosquito data stored, climate data at the locations and dates with mosquito observations were acquired.

The climate attributes used to develop the model were air temperature, humidity, cloud cover, pressure, and precipitation. Initially, an attempt was made to extract raw climate data from the SentinelHub API [34], which provides near real-time data from various satellites, e.g., Sentinels, Landsats. However, the low-level nature of the API made extracting information from raw pixel data time-consuming and error prone. Thus, another weather API—Storm Glass [35]—which provided both real time and historical data for a given point and time from various meteorological services (National Oceanic and Atmospheric Administration, Deutscher Wetterdienst, Météo-France, etc.) was identified. The API for the associated climate attributes at every point and time that was extracted earlier from the GLOBE and NEON datasets was queried. Storm Glass contained missing data for some required attributes at certain points and times—such points from our training set were removed. After deleting records with data gaps, the training set was composed of 15,838 mosquito observations from NEON and GLOBE Observer observations with associated climate data.

### 3.2. Machine Learning Approach

A decision tree is a technique used in supervised machine learning in which a label is generated based on decisions made at branches extending from nodes. Random Forest builds on this basic technique using the “Wisdom of the Crowd”, the idea that an aggregated prediction tends to be more accurate than an individual prediction. The use of several rather than individual predictors is called Ensemble Learning with an ensemble of decision trees being a Random Forest [36].

The process begins with a training dataset—the data that will be used to build the model. Then, in a process called bagging or bootstrap aggregation, the training dataset is randomly sampled with replacement and distributed to different predictors. In a Random Forest model, these predictors are decision trees. Each decision tree will be trained on their respective subset of the training data. Once trained, labels will be associated with certain combinations of decisions. Therefore, when new data arrives at the decision trees (usually from a user input), the model has already learned what label is associated with what combination of decisions, and a predicted label can be outputted for the new data based on what decisions are made for that data. Since Random Forest is based on a “majority vote” system, whatever value the majority of decision trees outputs is the value that is outputted by the model. In this study, binary classification will be used since the model will attempt to classify the data into two groups: presence and absence. For example, if absence is defined by 0 and presence by 1, and 11 out of 20 decision trees output a 0, the model output would be 0. A mathematical representation of this process of class prediction can be seen in Equation (1):

(1) $$f_{i_{i}}=\frac{\sum j:node\;j\;splits\;on\;feature\;i\;n_{j}}{\sum k\in \;all\;nodes\;ni_{k}\;}$$

The model learns the splits (threshold values for features that break a node into two branches) that yield a homogeneous (in terms of the label) subset of the training data. The dominant label at the end node will be the output when these combinations of decisions are made again following a user input. A model must also be able to split the data and determine the “quality” of that split. In binary classification, the optimum split would exactly divide one class from the other. To determine the quality of a split, Gini impurity is typically used in Random Forest.

Gini impurity can be defined more simply as the probability of incorrectly classifying an element. Gini values range from 0 to 0.5 and are calculated as shown in Equation (2). For example, a Gini impurity value of 0.5 says that the probability of incorrectly classifying an element would be 50%—the model would simply be “guessing” between 0 and 1. To avoid “guessing” and to maximize the assurance that the model’s prediction is correct, a decision tree in a Random Forest will continue to split the data until Gini impurity is as close as possible to 0. These procedures allow feature (e.g., climate variable) importance to be calculated:

(2) $$\begin{array}{c} G=1-\sum_{i=1}^{c}p{\left(i\right)}^{2}\\ C=\mathrm{Number}\;\mathrm{of}\;\mathrm{classes},\\ p(i)=\mathrm{Probability}\;\mathrm{of}\;\mathrm{selecting}\;\mathrm{class}\;i,\\ G=\mathrm{Gini}\;\mathrm{impurity} \end{array}$$

By determining how much each feature reduced Gini impurity on average, the most influential feature can be found [37]. Feature importance is calculated as shown in Equation (3) to determine which climate variable had the most influence on a mosquito presence or absence prediction:

(3) $$F\left(x\right)=\frac{1}{J}\sum_{j=1}^{J}c_{j_{full}}+\sum_{k=1}^{K}\left(\frac{1}{J}\sum_{j=1}^{J}\;\mathrm{contribution}{\;}_{j}\left(x,k\right)\right)$$

This is based on Hutchinson’s niche concept which states that environmental factors determine the space in which a species can live perpetually [38].

Matrix A in Equation (4) represents a set of linearly independent row vectors (an axis) that spans a space in which the ecological niche is defined. Another matrix will define the space where the species cannot survive. The Random Forest model attempts to determine the thresholds that divide the spaces defined by the matrix and the “absence matrix.” Based on these thresholds, the model will be able to predict whether a mosquito will be present or absent given certain climate inputs. However, for a more convenient user experience, the user does not have to provide the climate variable values. Instead, the user enters latitude and longitude coordinates. Corresponding Storm Glass data are then fed into the model and a prediction generated.

A row vector was assigned a 0 if it represented an absence point and 1 if it represented a presence point. The row vectors are also organized by latitude and longitude, but since location was not a variable in the model, it is excluded from the matrix above. The Random Forest algorithm itself was written in Python using the scikit-learn library. One thousand decision trees were generated with 90% of data to be used for training and 10% for testing. The model was trained to output a 0 or a 1 (two classes). All other parameters were set to their default value (the default parameter is Gini impurity rather than entropy for this library):

(4) $$\begin{array}{c} A=\left[\begin{array}{ccccc} a_{1} & b_{1} & c_{1} & d_{1} & e_{1}\\ a_{2} & b_{2} & c_{2} & d_{2} & e_{2}\\ \vdots & \vdots & \vdots & \vdots & \vdots \\ a_{n} & b_{n} & c_{n} & d_{n} & e_{n} \end{array}\right]\vec{Y_{1}}=\left\{a_{1},b_{1},c_{1},d_{1},e_{1}\right\}\;\mathrm{defines}\;\mathrm{one}\;\mathrm{mosquito}\;\mathrm{observation}\\ \vec{C_{1}}=\left\{a_{1},a_{2},a_{3}\cdots a_{n}\right\}\;\mathrm{defines}\;\mathrm{temperature}\\ \vec{C_{2}}=\left\{b_{1},b_{2},b_{3}\cdots b_{n}\right\}\;\mathrm{defines}\;\mathrm{humidity}\\ \vec{C_{3}}=\left\{c_{1},c_{2},c_{3}\cdots c_{n}\right\}\;\mathrm{defines}\;\mathrm{air}\;\mathrm{pressure}\\ \vec{C_{4}}=\left\{d_{1},d_{2},d_{3}\cdots d_{n}\right\}\;\mathrm{defines}\;\mathrm{precipitation}\\ \vec{C_{5}}=\left\{e_{1},e_{2},e_{3}\cdots e_{n}\right\}\;\mathrm{defines}\;\mathrm{cloud}\;\mathrm{cover}\; \end{array}$$

### 3.3. Edge Computing

An IoT device capable of collecting localized sensor data and outputting a threat level entirely on the edge was configured as a potential solution for areas with little cloud connectivity. GPS data from a smartphone were used to extract latitude and longitude data, which was then sent to the microcomputer via Bluetooth/USB C. To analyze and compute on the edge in a cost-effective manner, a relatively high-performance Raspberry Pi 4 Model B was chosen.

**Figure 2:**
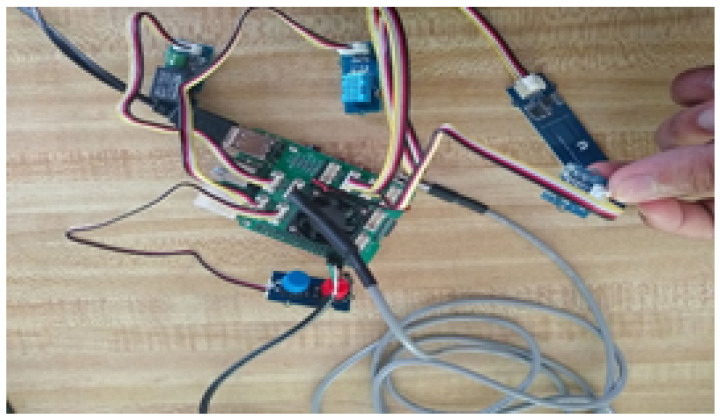
Mosquito edge framework configured with sensors and PC.

To collect sensor data, the prebuilt Grove Smart Agriculture Kit from the agriculture industry was leveraged as using existing tools allows for greater flexibility and integration with preexisting frameworks. The kit provided access to several external Grove sensors that were assembled onto the Raspberry Pi 4 Model B as seen in Figure 2. These sensors collect air temperature, humidity, soil moisture, soil temperature, UV light, IR light, and visible light data.

This climate data as well as the exact date and GPS coordinates of its collection are recorded by the device. The data are then used as input to run a TensorFlow Lite model (a low footprint format) on the device, removing the need for a PC entirely and truly moving our device to the edge. This localization of data collection and analysis allows for increased speed and removes the need for Wi-Fi access.

## 4. Results

The Hutchinson matrix that defines the ecological niche of a mosquito was implemented as the training data for the model. This is shown in Table 1.

Then, the training dataset was loaded onto the Raspberry Pi 4 as a CSV file. Sensors that were used include a temperature and humidity sensor, and GPS coordinates were sent from a smartphone via USB C. When the model was run on the edge in Los Gatos, CA, with the inputs being the data collected from the climate sensors attached to the device, a threat level of 42.5% was successfully outputted with an accuracy was 86% as shown in Table 2. This is very accurate considering the vast number of variables that cannot be accounted for in an ecological niche model. The confusion matrix supports this accuracy rating—there is a high number of true positives (772) and true negatives (588) while there is a relatively small number of false positives (108) and false negatives (116).

The relative feature importance was calculated for all five climate variables and can be seen in Table 3. The data suggest that temperature has the strongest association with threat level followed by humidity, pressure, cloud cover, and precipitation in order of weakening association.

Another objective of the Random Forest model was to produce a “threat level” or the probability of a mosquito presence point. This number can be attained by calculating the number of decision trees out of the 1000 in the forest that voted for a presence (“1”) based on the user location input and its corresponding climate variables. This model can be run for any year after 2017 (limited by a lack of historical data in the Storm Glass API, which will soon be remedied) up until the present and at any location on Earth. After running the model hundreds of times, aggregate threat outputs were correlated with the climate variables.

**Figure 3:**
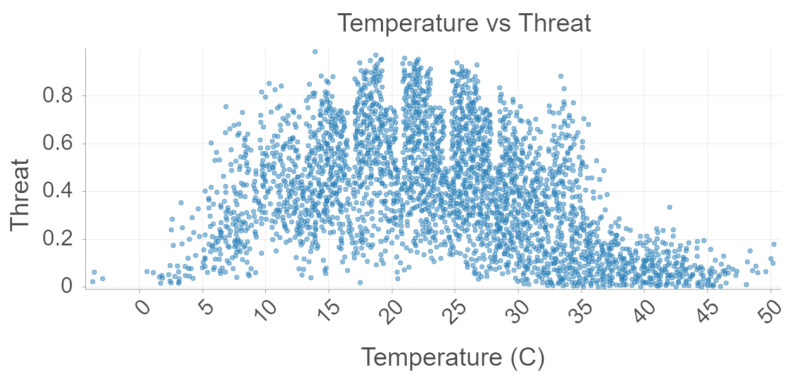
Temperature and Threat analysis using Random Forest.

In Figure 3, temperature vs. threat level (probability of a presence point) is plotted for the 2021 data. The relationship between these two variables is nonlinear. As temperature increases, threat level increases as well. However, past a threshold value of about 28 degrees Celsius, the threat rapidly decreases. This is most likely due to mosquitoes’ aversion to very hot, dry climates such as the Sahara region in favor of fairly hot, humid regions. Once again, the similarities between the relationships seen in our model and ecological phenomena is a testament to the model’s high accuracy.

**Figure 4:**
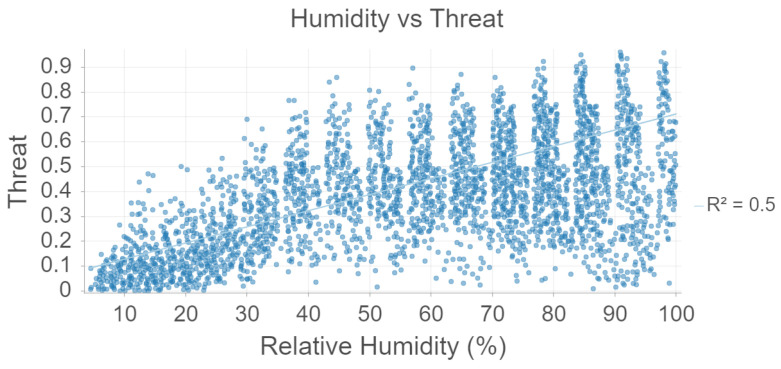
Humidity and Threat analysis using Random Forest.

Figure 4, humidity vs. threat level is plotted for the 2021 data. There is a moderate, linear association between humidity and threat. This is consistent with Table 3 which suggests a moderate association and the ecological literature which suggest that mosquitoes prefer humid climates.

**Figure 5:**
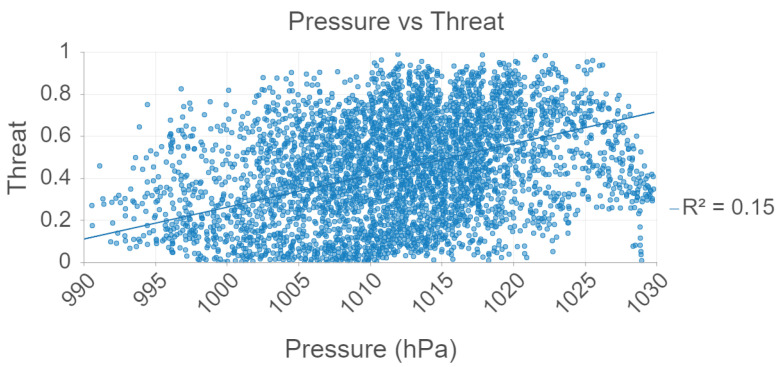
Pressure and Threat analysis using Random Forest.

Figure 5, pressure vs. threat level is plotted for the 2021 data. There is a weak, linear association between pressure and threat. However, this finding may be perhaps insignificant since there is no ecological support for why mosquitoes would prefer regions with high air pressure.

**Figure 6:**
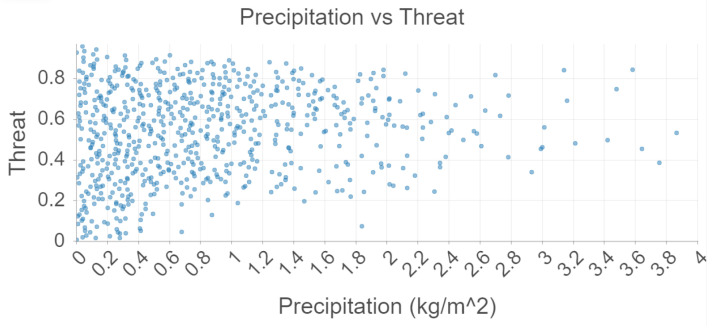
Precipitation and Threat analysis using Random Forest.

There is no association between precipitation and threat as seen in Figure 6. This aligns with Table 3, which suggests a weak correlation. This is also further support to the belief that mosquitoes are attracted to standing water rather than precipitation itself. Future studies should examine the relationship between precipitation and threat days after the precipitation has already occurred.

**Figure 7:**
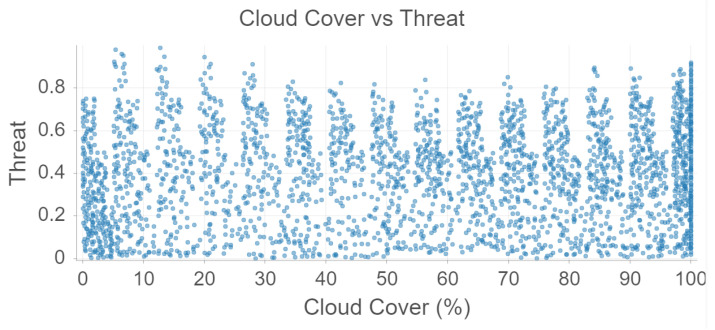
Cloud Cover and Threat analysis using Random Forest.

Cloud cover is somewhat correlated to temperature and precipitation, but there seems to be no relationship between cloud cover and threat level according to Figure 7.

## 5. Discussion

A neural network, linear regression, and logistic regression were run on the aforementioned data. The neural network model has an accuracy score of 74% while the logistic regression performs at 67%. A comparable accuracy score cannot be calculated for a continuous target variable, but the linear regression has a coefficient of determination of 0.16, suggesting that the linear model is not accurate nor is it suitable for classification. All three algorithms performed at a lower accuracy than Random Forest. This reinforces past literature, which has suggested that niche prediction is best modeled by an ensemble approach.

Temperature clearly has the most influence on the model output—a result that is consistent with ecological wisdom that suggests mosquito presence and absence is strongly correlated with high and low temperatures respectively. Precipitation has a relatively low impact on the model, which is consistent with the ecological literature that suggests that mosquitoes are attracted to standing water rather than precipitation itself. Mosquitoes prefer humid environments—this is consistent with the relative importance results. We are able to perform this analysis in real time through the running of the edge device model and collecting of local data from the device.

As with any research, our model might have been affected by errors. For one, citizen science data are not as accurate as data collected by trained scientists. As our mosquito dataset did not contain information about specific mosquito species, it is possibly biased towards the more common mosquito species (e.g., Aedes, Culex, and Anopheles), potentially leading to excessive generalization and low accuracy for predictions in areas with less common mosquitoes with different environmental niches.Furthermore, resource intensive AI implementations on the edge on low power IoT devices can lead to high latency and low accuracy.

While our model may use an ensemble approach similar to that of other mosquito tracking software, our model differs through its use in variables and scale. Specifically, other software focuses singularly on a specific genus (and often species) of a mosquito that is concentrated in a particular area of the world—our software by contrast focuses on all mosquitoes in all parts of the world. Additionally, our software uses precipitation, cloud cover, atmospheric pressure, temperature, and humidity to predict mosquito hotspots, while other software largely uses land cover data coupled with temperature and precipitation. This in part is what leads to a lower accuracy of 86%, whereas other software has a significantly higher accuracy.

Other research papers have acknowledged different variables that can affect certain mosquito populations such as *Culiseta annulata*, *Anopheles claviger* and *Ochlerotatus punctor* [14]. Species of mosquitoes are affected by different combinations and variables’ importance to mosquito populations have changed, suggesting that not all mosquito’s species are affected equally by changes in certain climate variables. Ultimately, researchers were able to achieve a 99% accuracy model using the random forest framework to predict the spatial distribution of *Anopheles Claviger*.

## 6. Conclusions

A combination of temperature, humidity, atmospheric pressure, precipitation, and cloud cover data were used to create a model with 86% accuracy. There was a direct correlation between moderate, linear association between humidity and threat, but a weak, linear association between pressure and threat.

Our findings reinforce the current theories that mosquitoes are heavily influenced by their environment, and although there are approximately 3500 species of mosquitoes, all desire a certain range of climate conditions. Our study has taken thousands of data points from NEON, Storm Glass API, and the GLOBE Observer, and coupled with our accuracy, our model makes statistically significant findings.

The accuracy of the model can be increased by adding more environmental features, such as normalized vegetation difference index and human population density. Our model could also become more accurate by tailoring it to specific mosquito species rather than treating all mosquitoes as a single species with the same environmental preferences. This would allow scientists to predict the threat level for a particular species with more confidence, especially for less common species. We are also working on integrating our model with the Find Your Invasive framework.

For the future, there are plans to pair the edge device with a mosquito trap and directly capture mosquito data with computer vision techniques. This would be an autonomous system that can be setup in remote areas without manual oversight, increasing efficiency dramatically.

## Figures and Tables

**Table 1:** First 5 rows of training data.

	Temp (°C)	Rel. Humidity (%)	Pressure (hPa)	Precipitation (kg/m^3^)	Cloud Cover (%)	Presence
0	15.67	87.87	1023.32	0.48	96.67	1
1	15.67	87.87	1023.32	0.48	96.67	1
2	15.67	87.87	1023.32	0.48	96.67	1
3	16.19	83.03	1024.08	0.24	97.33	1
4	16.19	83.03	1024.08	0.24	97.33	1

**Table 2:** Random Forest model performance.

	Precision	Recall	F1-Score	Support
0	0.87	0.88	0.87	888
1	0.84	0.84	0.84	784
acccuracy			0.86	1584
macro avg	0.86	0.86	0.86	1584
weighted avg	0.86	0.86	0.86	1584

**Table 3:** Random Forest model feature importance.

Feature	Score
Temperature	0.33783
Humidity	0.21056
Pressure	0.18738
Precipitation	0.12785
Cloud Cover	0.13718
